# From Diagnosis to Behaviour Change: Applying the Health Action Process Approach to Smoking Cessation After Head and Neck Cancer

**DOI:** 10.3390/bs16020293

**Published:** 2026-02-18

**Authors:** Anaëlle Préaubert, Agnès Dupret-Bories, Emilien Chabrillac, Florence Sordes, Patrick Raynal

**Affiliations:** 1 Centre d’Etudes et de Recherches en Psychopathologie et Psychologie de la Santé, Universités de Toulouse, EA 7411, 31058 Toulouse, France; 2 Département de Chirurgie de la Tête et du Cou, Institut Universitaire du Cancer de Toulouse-Oncopole, Centre Hospitalo-Universitaire de Toulouse, Universités de Toulouse, 31059 Toulouse, France; 3 Département de Chirurgie Cervico-Faciale, Institut Universitaire du Cancer de Toulouse-Oncopole, Oncopole Claudius Regaud, Universités de Toulouse, 31059 Toulouse, France

**Keywords:** Health Action Process Approach, smoking cessation, health behavior change, self-efficacy, action control, head and neck cancer, longitudinal study

## Abstract

Smoking cessation after a cancer diagnosis is a key determinant of prognosis, yet the psychological mechanisms underlying cessation remain poorly understood. Building on a recently validated Health Action Process Approach (HAPA) scale, this study examined whether baseline HAPA constructs predicted short-term smoking cessation and tobacco dependence in patients with head and neck cancer. Eighty-nine patients completed assessments at diagnosis (T0) and one-month follow-up (T1). Six HAPA constructs were measured at T0: Risk Perception, Outcome Expectancies, Recovery Self-Efficacy, Behavioral Intention, Coping Planning, and Action Control Efficacy. Smoking outcomes at T1 included cigarette dependence (CDS-12) and smoking status. Hierarchical linear regression showed that sociodemographic and clinical variables did not predict dependence, whereas adding HAPA constructs significantly improved prediction (Δ*R*^2^ = 0.28, *p* < 0.001). Higher Risk Perception and Outcome Expectancies were associated with greater dependence, while logistic regression identified Action Control Efficacy as the only independent predictor of smoking cessation. These findings provide the first longitudinal evidence supporting the application of the HAPA framework to smoking cessation after cancer diagnosis and underscore the critical role of volitional processes in early cessation. Targeting action control may therefore enhance the effectiveness of smoking cessation interventions in oncology settings.

## 1. Introduction

Head and neck (H&N) cancers, encompassing tumors of the oral cavity, pharynx, and larynx, are the sixth most common cancers worldwide, representing around 6–7% of all malignancies (Gormley et al., 2022; Li et al., 2023; Sung et al., 2021). Tobacco use is consistently identified as a major etiological factor, with numerous studies highlighting its strong contribution to the development of H&N malignancies (Cleere et al., 2024; Dai et al., 2022; Simmons et al., 2020; J. Smith et al., 2021). Smoking is responsible for the majority of cases, with tobacco, often in combination with alcohol, accounting for 70–80% of H&N cancers and up to 89% of laryngeal cancers (Gormley et al., 2022; Torrens et al., 2025).

Persistent tobacco use following a diagnosis of H&N cancer has been consistently linked to poorer clinical outcomes (Nagappa et al., 2023; J. Smith et al., 2019). Conversely, smoking cessation after diagnosis has been associated with increased overall survival (Khalifeh et al., 2024; Krutz et al., 2022; Lee et al., 2023; Ma et al., 2022; von Kroge et al., 2020). Continued smoking negatively impacts prognosis by diminishing the effectiveness of cancer therapies, particularly radiotherapy (Chen et al., 2011; Krutz et al., 2022; Lee et al., 2023; Ma et al., 2022) and is also a risk factor for postoperative complications such as impaired wound healing (Crippen et al., 2019; Fan Chiang et al., 2023; Garip et al., 2021). It also heightens the likelihood of both acute and long-term treatment-related complications. For example, smoking during radiotherapy significantly increases the risk and severity of acute toxicities such as oral mucositis (Bergman et al., 2022; Dewi et al., 2022; Perdyan & Jassem, 2022; Tan et al., 2025), as well as late complications like osteoradionecrosis, which most often affects the mandible and is primarily associated with high radiation doses and poor oral health (Céspedes-Ajún et al., 2022; Lang et al., 2022; Yang et al., 2024). Additionally, continuing to smoke after a diagnosis of H&N cancer more than doubles the risk of recurrence or disease progression compared to quitting (Caini et al., 2025; Jassem, 2019; Mohebbi et al., 2025).

Although the harmful effects of continued smoking after a cancer diagnosis are well established, a notable proportion of patients with H&N cancer persist in smoking. Estimates of smoking continuation vary widely across studies, ranging from around 10% to nearly 60% (Burris et al., 2015; McCarter et al., 2022; Penfold et al., 2018; Underwood et al., 2012; Van Heest et al., 2022; Verro et al., 2024). Longitudinal research further shows that these rates remain high over time: between 40% and 60% of patients continue smoking six months after diagnosis, with 40–45% still smoking at 12 to 24 months (Nagappa et al., 2023; Paul et al., 2019; Van Heest et al., 2022). Such persistence highlights the chronic nature of tobacco dependence in this population and underscores the need to identify the sociodemographic, clinical, and psychosocial factors that hinder cessation.

Understanding and addressing the complex factors that influence smoking behavior, particularly after a cancer diagnosis, could require the support of well-established theoretical frameworks. Among these, the Health Action Process Approach (HAPA) offers a socio-cognitive model that captures the dynamic nature of health behavior change (Schwarzer, 2008; Schwarzer et al., 2011). The HAPA model is structured around two key phases: a motivational phase, during which individuals evaluate the risks of their behavior, consider the potential benefits of change, and build self-efficacy to initiate action; and a volitional phase, which involves translating intention into behavior through concrete planning (e.g., action and coping planning), sustained self-regulation, and the management of barriers. This dual-phase approach not only helps clarify the psychological mechanisms underlying health behavior but also provides practical guidance for designing interventions that are responsive to an individual’s position along the change process. (Schwarzer, 2008; Schwarzer et al., 2011).

The HAPA model has been widely used to explain and support changes in various health-related behaviors. Its applicability has been demonstrated in domains such as healthy eating (Jiang et al., 2024; Parkinson et al., 2023; Radtke et al., 2014; Renner et al., 2008), oral hygiene (Araújo et al., 2020; Moghaddam et al., 2025; Van Nes et al., 2024; S. R. Smith et al., 2024), diabetes management (Döbler et al., 2018; MacPhail et al., 2014; Ranjbaran et al., 2020, 2022), and physical activity promotion (Barg et al., 2012; Lindblom & Hägglund, 2024; Maghsoodlo et al., 2024; Zhou et al., 2021). The model has also proven useful in the context of oncology, particularly for designing interventions aimed at increasing physical activity among cancer patients (Hardcastle et al., 2022; Matsui, 2024; Maxwell-Smith et al., 2019; Paxton, 2016; Sequeira et al., 2022). Although several studies have investigated the use of the HAPA framework in smoking cessation (Berli et al., 2015; Lin et al., 2024; Scholz et al., 2009; Schwarzer & Luszczynska, 2008; Williams et al., 2011), its specific application to smoking behavior change following a cancer diagnosis has not yet been explored. This gap in the literature highlights the need to better understand the psychosocial determinants of smoking cessation during this critical period and to design tailored interventions. Although the HAPA model provides a relevant framework for guiding such interventions, evidence of its predictive validity in cancer populations is still limited, and its application to tobacco use after diagnosis remains largely unexplored.

In a previous study (Préaubert et al., *manuscript under review*), we developed and conducted a psychometric assessment of a HAPA-based scale for smoking cessation in patients recently diagnosed with H&N cancer. While the scale demonstrated good psychometric properties, its predictive value has yet to be tested in a longitudinal framework. The aim of the present study was to evaluate whether HAPA constructs assessed shortly after diagnosis could predict smoking behavior one month later. More specifically, we sought to determine whether motivational and volitional variables predicted (1) the level of cigarette dependence and craving at follow-up, and (2) smoking cessation status (abstinence vs. continued smoking). Guided by the HAPA framework, we formulated an a priori, theory-driven hypothesis that motivational and volitional HAPA constructs assessed at diagnosis would be prospectively associated with smoking outcomes at follow-up. This study contributes to the growing body of research on theory-based smoking cessation models by exploring the utility of HAPA constructs in predicting short-term behavioral outcomes among patients with H&N cancer.

## 2. Materials and Methods

### 2.1. The Participants and Procedures

Data were collected through an in-person survey conducted at a specialized cancer hospital (University Cancer Institute of Toulouse-Oncopole of Toulouse, France), within the Otolaryngology consultation department. Participant recruitment, carried out by surgeons at the Oncopole, began in November 2023 and concluded in February 2025, using a convenience clinical sampling strategy. To be eligible, participants had to be adults, report a current tobacco consumption of at least two cigarettes per day, and have received a recent diagnosis of H&N cancer. A total of 134 participants were initially included at cancer diagnosis announcement (T0). Among them, 89 completed both T0 and one-month follow-up (T1) assessments and were retained for the final analyses. The survey was administered in paper format, and responses were anonymized to ensure confidentiality. To allow for data matching across time points while preserving anonymity, each participant was assigned a unique identification code at T0, which they were instructed to provide again when completing the T1. The completeness and consistency of the questionnaires were verified upon submission. No financial compensation was provided for participation. The study received ethical approval from a local ethics committee (Comité de Protection des Personnes Sud-Ouest et Outre-Mer II, reference number SI 23.01630.000204, national number 2023-A00513-42) and from the Data Protection Officer of the University of Toulouse (assessment number R-202304040950). All participants provided written informed consent before completing the survey.

### 2.2. Measures

#### 2.2.1. Personal Information

Personal information was collected, including gender, age, marital status, socio-occupational category, type of cancer diagnosis and treatment, tumor stage, and the number of cigarettes smoked per day at T0 and at T1.

#### 2.2.2. HAPA-Based Smoking Cessation Measure for H&N Cancer Patients

A smoking cessation scale was specifically developed for H&N cancer patients based on the HAPA (HAPA; Schwarzer, 2008). The original version included 27 items covering eight theoretical constructs of the HAPA model. However, an exploratory factor analysis conducted in a previous study (Préaubert et al., *manuscript under review*) supported a six-factor structure, in which Action Self-efficacy, Maintenance Self-Efficacy, and Action Planning were grouped under a single latent dimension labelled Action Control Efficacy. The final version of the scale thus includes three constructs categorized as motivational (Risk Perception, Outcome Expectancies, and Behavioral Intention), whereas the other three were classified as volitional (Recovery Self-Efficacy, Coping Planning, and Action Control Efficacy). Example items include “If I quit smoking, I will improve the effectiveness of my treatments and increase my chances of recovery” (Outcome Expectancies) and “Even if I relapse and smoke for a few days, I am confident that I will be able to quit again” (Recovery Self-Efficacy). The items were rated using either 4-point or 7-point Likert scales, depending on the nature of the construct, in accordance with Schwarzer’s recommendations (Schwarzer, 2008). Higher scores indicate stronger endorsement of the corresponding psychological determinant of smoking cessation. In the validation study, all subscales demonstrated satisfactory to excellent internal consistency, with Cronbach’s α ranging from 0.71 to 0.96. Further details regarding item content, factor structure, and psychometric properties are reported in the original validation study (Préaubert et al., *manuscript under review*).

#### 2.2.3. Cigarette Dependence Scale

Cigarette dependence was measured using the Cigarette Dependence Scale (CDS-12) (Etter et al., 2003), a self-report tool assessing behavioral, cognitive, and emotional aspects of tobacco dependence. The scale comprises 12 items with varied formats, including Likert-type responses, multiple-choice questions (e.g., daily cigarette use, time to first cigarette), and a visual analog item rating perceived dependence from 0 (not at all dependent) to 100 (extremely dependent). A sample item is: “I feel stressed at the idea of running out of cigarettes”. Higher scores reflect greater dependence. The CDS-12 has demonstrated good reliability in previous studies (Cronbach’s alpha ≥ 0.84) (Etter et al., 2003). In the current sample, internal consistency was also high (Cronbach’s alpha = 0.92).

#### 2.2.4. Tobacco Craving Questionnaire

Tobacco craving was assessed using the French Tobacco Craving Questionnaire (FTCQ-12) (Berlin et al., 2005), a validated instrument designed to assess the intensity and multidimensional nature of craving in individuals who smoke. The questionnaire includes 12 items covering emotional, cognitive, and behavioral dimensions of craving. Each item is rated on a 7-point Likert scale, ranging from 1 (not at all true) to 7 (completely true). A sample item is: “If there were a cigarette here in front of me, it would be very difficult not to smoke it”. Some items are negatively worded and reverse-coded so that higher scores uniformly reflect greater craving intensity. In the original validation study, internal consistency for the four subscales of the French version ranged from acceptable to good, with Cronbach’s alpha coefficients between 0.66 and 0.83 (Berlin et al., 2005). In the present study, internal consistency of the total score was excellent (Cronbach’s alpha = 0.87).

### 2.3. Data Preparation and Statistical Analysis

Of the 134 participants initially included at T0, 45 were excluded from the final analyses due to excessive missing data, defined as having more than 5% of items left unanswered across the entire questionnaire. Although the completeness and consistency of the questionnaires were checked upon submission, a more stringent data cleaning procedure was later applied before statistical analysis. The final sample thus included 89 participants who completed both assessment points (T0 and T1). For the remaining sporadic missing data, Little’s MCAR test indicated that data were missing completely at random, *χ*^2^(11) = 19.89, *p* = 0.047. Univariate outliers were identified using z-scores, with absolute values above 3.29 considered extreme. Multivariate outliers were assessed using Mahalanobis distance, with a significance threshold set at *p* < 0.001 (Tabachnick & Fidell, 2019). No significant outliers were detected. Normality assumptions were assessed through skewness and kurtosis. While most variables fell within the acceptable range of −1 to +1, kurtosis exceeded this threshold for the CDS-12 at T1 (−1.26). The FTCQ-12 (0.62) fell within the acceptable range. All statistical analyses were performed using IBM SPSS Statistics version 29.

## 3. Results

### 3.1. Sample Characteristics and Descriptive Statistics

The final sample consisted of 89 individuals with a mean age of 59.6 years (*SD* = 10.4). Most participants identified as men (*n* = 66, 74.2%), while 23 participants (25.8%) identified as women. In terms of marital status, 48 participants (53.9%) were single and 41 (46.1%) were in a relationship. Regarding socio-professional status, 53 participants (59.6%) were inactive (retired or unemployed), 23 (25.8%) were employees or manual workers, 7 (7.9%) belonged to executive or intellectual professions, and 6 (6.7%) were self-employed (including entrepreneurs and craftsmen). The most common diagnosis was oral cavity cancer (*n* = 34, 38.2%), followed by oropharyngeal cancers (*n* = 20, 22.5%), laryngeal cancers (*n* = 19, 21.3%), hypo- and nasopharyngeal cancers (*n* = 11, 12.4%), and other H&N cancers (*n* = 5, 5.6%). Most participants had advanced-stage tumors (Stage III/IV: 82.5%), and surgery alone was the most frequently reported treatment (*n* = 44, 40.7%), followed by radiotherapy alone (19.4%), surgery and radiotherapy combined (18.5%), chemoradiotherapy (15.7%), and other treatments (5.6%). At T0, most participants reported smoking between 2 and 10 cigarettes per day (60.7%), while 30.3% smoked 11–20, and 9% smoked more than 20. One month later (T1), 44.9% had stopped smoking, 31.6% smoked between 2 and 10 cigarettes daily, 20.2% smoked 11–20, and 3.3% continued to smoke more than 20 cigarettes daily. Other demographic and clinical characteristics of the sample are detailed in Table 1.

### 3.2. Associations Between HAPA Constructs and Smoking Outcomes

A Pearson correlation analysis was conducted to examine both motivational and volitional HAPA constructs assessed at T0 and smoking-related variables measured at T0 and T1 (Table 2). Recovery Self-Efficacy was negatively associated with cigarette dependence both at T0 (*r* = −0.39, *p* < 0.01) and at T1 (*r* = −0.34, *p* < 0.01), as well as with tobacco craving at T0 (*r* = −0.36, *p* < 0.01) and at T1 (*r* = −0.29, *p* < 0.01). These associations indicate moderate negative correlations, except for the latter, which was low to moderate. Coping Planning was also negatively correlated with cigarette dependence at T1 (*r* = −0.34, *p* < 0.01), and with tobacco craving at T0 (*r* = −0.26, *p* < 0.05) and T1 (*r* = −0.27, *p* < 0.05), reflecting moderate relationships. Similarly, Action Control Efficacy indicates inverse associations with cigarette dependence at T0 (*r* = −0.35, *p* < 0.01) and T1 (*r* = −0.39, *p* < 0.01), as well as with tobacco craving at T0 (*r* = −0.39, *p* < 0.01) and T1 (*r* = −0.36, *p* < 0.01). These coefficients consistently point to moderate negative correlations. Overall, higher Recovery Self-Efficacy, Coping Planning, and Action Control Efficacy at diagnosis were linked to lower levels of cigarette dependence and craving, both concurrently and prospectively.

### 3.3. Binomial Logistic Regression Predicting Smoking Status at T1

To identify which psychological constructs assessed at T0 predicted smoking cessation status at T1, a binomial logistic regression analysis was conducted. The outcome variable was smoking status at T1, coded as 0 = continued smoking and 1 = smoking cessation. The six HAPA constructs measured at T0, namely Risk Perception, Outcome Expectancies, Recovery Self-Efficacy, Behavioral Intention, Coping Planning, and Action Control Efficacy, were simultaneously entered in the model as independent variables. The model significantly distinguished between smokers and non-smokers, *χ*^2^(6) = 20.19, *p* = 0.003, indicating that the predictors reliably explained smoking cessation status. However, among all variables included in the model, Action Control Efficacy emerged as the only significant independent predictor of smoking cessation (*B* = −0.095, *p* = 0.02); all other predictors were non-significant (*B* range= −0.058 to 0.055, *p* range = 0.28 to 0.92). This suggested that participants scoring higher on Action Control Efficacy, reflecting greater confidence in their ability to plan, initiate, and maintain smoking cessation behaviors, were more likely to have quit smoking at follow-up.

### 3.4. Sociodemographic, Clinical, and HAPA Constructs of Smoking Dependence

To examine whether psychological and motivational factors assessed at T1 could predict subsequent levels of tobacco dependence, a hierarchical multiple linear regression analysis was conducted using CDS-12 scores at T1 as the dependent variable. This analysis aimed to test the added predictive value of HAPA constructs, as independent variables, beyond sociodemographic and clinical characteristics. In the first step, sociodemographic and clinical characteristic variables, including gender, age, marital status, socio-professional category, cancer diagnosis, treatment, and tumor stage, were entered. This model was not significant, *F*(7, 81) = 0.76, *p* = 0.62, indicating that these background variables did not explain a significant proportion of the variance in cigarette dependence scores (Table 3). In the second step, the six HAPA constructs measured at T0 were included. The full model reached significance, *F*(13, 73) = 2.74, *p* = 0.004, with the HAPA constructs accounting for an additional 28.2% of the variance (Δ*R*^2^ = 0.282, *p* < 0.001). Two predictors emerged as significant: Risk Perception (*β* = 0.34, *t* = 2.92, *p* = 0.005) and Outcome Expectancies (*β* = 0.26, *t* = 2.02, *p* = 0.047), suggesting that higher perceived risk and more favorable expectations about smoking cessation were associated with greater tobacco dependence at T1. Overall, the hierarchical regression indicated that adding HAPA constructs significantly improved the prediction of tobacco dependence at T1 beyond sociodemographic and clinical variables.

## 4. Discussion

The present study examined the predictive validity of a HAPA-based scale for smoking cessation in patients recently diagnosed with H&N cancer. While this instrument had previously shown satisfactory psychometric properties (Préaubert et al., *manuscript under review*), its ability to prospectively predict smoking-related outcomes had not yet been established. Consistent with this objective, we investigated whether motivational and volitional constructs of the HAPA assessed shortly after diagnosis could account for subsequent levels of cigarette dependence and craving, as well as smoking cessation status one month later. In line with the general and theory-driven nature of our a priori hypothesis, the present findings indicate that HAPA constructs do not contribute uniformly to short-term smoking outcomes, highlighting the predominant role of volitional processes, particularly Action Control Efficacy, in early smoking cessation after diagnosis.

The correlation analyses conducted in this study revealed significant associations between several volitional HAPA constructs and smoking-related outcomes. In particular, Recovery Self-Efficacy, defined as confidence in one’s ability to regain control after smoking cessation relapse (Schwarzer, 2008), was negatively associated with both cigarette dependence and tobacco craving at T0 and T1. This indicated that patients who reported greater confidence in recovering from relapse tended to show lower levels of dependence and craving over time. This finding resonates with prior research identifying self-efficacy as a central determinant of addictive behavior regulation and smoking cessation outcomes (Gwaltney et al., 2005; Scholz et al., 2009; Shiffman et al., 2000).

Coping Planning, defined as the process of anticipating potential obstacles and preparing strategies to overcome them (Schwarzer, 2008), was also negatively correlated with dependence and craving. This suggested that Coping Planning may serve as a protective factor, by equipping individuals with strategies to manage high-risk relapse situations that are particularly associated with smoking behavior and resist urges. This interpretation is consistent with the HAPA framework, which emphasizes planning as a key volitional mechanism bridging the intention–behavior gap, referring to the frequent observation that strong intentions to change do not always translate into actual behavior (Gollwitzer & Sheeran, 2009; Schwarzer, 2008). In oncology populations, where stress and treatment-related side effects can exacerbate smoking urges, coping planning may thus represent an especially valuable psychological resource (Antoni et al., 2023; Macía et al., 2020).

To examine which baseline HAPA constructs could prospectively predict smoking cessation at one month, we conducted a logistic regression analysis. Results revealed that only Action Control Efficacy significantly predicted cessation status at T1. This construct, which integrates confidence in one’s ability to initiate and maintain abstinence (Action and Maintenance Self-Efficacy) with the capacity to translate intentions into concrete plans (Action Planning) (Préaubert et al., *manuscript under review*; Schwarzer, 2008), reflects a broad volitional resource for self-regulation. Participants reporting higher Action Control Efficacy were more likely to have quit at follow-up, whereas Risk Perception, Outcome Expectancies, Recovery Self-Efficacy, Behavioral Intention, and Coping Planning did not independently predict cessation. Within the HAPA framework, Risk Perception, Outcome Expectancies, and Behavioral Intention are primarily involved in intention formation, while volitional constructs support the enactment of behavior change (Schwarzer, 2008). In patients newly diagnosed with H&N cancer, motivation to quit may already be strongly heightened at diagnosis, limiting the additional predictive value of motivational constructs, while making volitional resources such as Action Control Efficacy critical for translating this motivation into abstinence (Howren et al., 2013; Mäkitie et al., 2024). By jointly capturing self-efficacy and planning, Action Control Efficacy reflects mechanisms consistently identified as essential for successful behavior change and maintenance (Lippke et al., 2009; Schwarzer et al., 2010; Zhang et al., 2019), which may explain its unique predictive value in the present study and is consistent with evidence linking greater perceived control to better cessation outcomes (Rahmawati et al., 2022; Schnoll et al., 2011; Yzer & van den Putte, 2014). Within this framework, Coping Planning may play a downstream or secondary role, becoming effective only once individuals feel capable of actively monitoring and regulating their behavior. Action Control Efficacy may therefore partially subsume processes typically attributed to Coping Planning, particularly in the early phase of cessation and within a short-term follow-up context, where self-regulatory confidence and action initiation are paramount.

The hierarchical regression examined whether psychological and motivational factors at T0 predicted tobacco dependence at T1 beyond sociodemographic and clinical variables. Age, gender, marital status, socio-professional category, cancer diagnosis, treatment, and tumor stage did not significantly explain variance, suggesting that background factors alone have limited predictive value for short-term dependence. This aligns with evidence that psychological processes such as distress, coping, and psychological flexibility exert a more dynamic influence on smoking outcomes (Fluharty et al., 2017; Guimond et al., 2017; Hakulinen et al., 2015; Kock et al., 2022; Lespine et al., 2025). Adding HAPA constructs significantly improved the model, explaining the 28% additional variance. Risk Perception, defined as awareness of health risks associated with continued smoking, and Outcome Expectancies, referring to beliefs about the benefits of quitting or harms of smoking (Schwarzer, 2008), emerged as significant, but in an unexpected direction: higher scores were associated with greater dependence. This counterintuitive pattern may reflect anxiety-driven smoking or cognitive dissonance processes, whereby highly dependent patients are more aware of the risks and benefits associated with smoking cessation, yet experience greater psychological distress and craving that hinder behavioral change. In this context, heightened risk perception and outcome expectancies may signal motivation without sufficient volitional resources to translate awareness into action, as supported by evidence in cancer populations linking higher distress and craving to poorer cessation outcomes despite strong motivation (Guimond et al., 2017; Streck et al., 2021).

This paradox is consistent with addiction research showing that highly dependent smokers often recognize risks and benefits but struggle to translate this awareness into action. Both neurobiological and psychological models attribute this gap to craving, withdrawal, and impaired self-regulation, which weaken the link between motivation and behavior (Gladwin et al., 2011; Koob & Volkow, 2016; Köpetz et al., 2013). Although a cancer diagnosis is frequently described as a “teachable moment” that heightens motivation and risk awareness (Frazer et al., 2022; McBride, 2003), motivation alone appears insufficient when dependence is strong, as compulsive processes can override quit intentions (Andrade et al., 2012; Tiffany & Conklin, 2000). This mechanism may explain why many patients continue smoking despite diagnosis, despite the well-documented benefits of cessation for survival and recurrence (Paul et al., 2019; von Kroge et al., 2020). Overall, elevated Risk Perception and Outcome Expectancies among highly dependent patients likely reflect a motivation–action gap rather than readiness to quit, underscoring the need for interventions targeting volitional resources such as Recovery Self-Efficacy, Coping Planning, and Action Control Efficacy.

From a clinical perspective, these findings suggest that smoking cessation support for H&N cancer patients should go beyond interventions that merely raise risk awareness or outcome salience. Although a cancer diagnosis often acts as a powerful “teachable moment” that enhances motivation to quit (Frazer et al., 2022; Jassem, 2019; McBride, 2003), motivation alone is frequently insufficient in the context of strong nicotine dependence, where craving and compulsive processes can override quit intentions (Andrade et al., 2012; Tiffany & Conklin, 2000). These results highlight the importance of targeting volitional resources, particularly Action Control Efficacy, to support the translation of motivation into sustained abstinence. This may be achieved through interventions emphasizing structured action planning, coping with high-risk situations, and relapse recovery skills (Lippke et al., 2009; Schwarzer et al., 2010). Such approaches appear especially relevant for H&N cancer patients, who commonly present with high nicotine dependence and elevated relapse risk (Conlon et al., 2020; Naresh et al., 2020), and may ultimately improve both cessation outcomes and quality of life.

Several limitations should be acknowledged. First, although 134 participants were initially included at baseline, the final analytical sample was reduced to 89 due to the exclusion of participants with excessive missing data following a stringent data-cleaning procedure, which may have limited representativeness. The sample size was therefore modest (N = 89), which may limit statistical power. However, recruitment and retention in longitudinal studies of patients with H&N cancer are particularly challenging due to high symptom burden, intensive treatments, and psychosocial vulnerability, especially in smoking cessation research where stigma and relapse are common (Mao & Bottorff, 2017; Mody et al., 2021). Second, smoking status was self-reported, which may introduce social desirability bias, particularly in oncology settings where continued smoking is stigmatized (Auriol et al., 2025; Clark et al., 2016). Although self-reports of smoking are generally reliable (Bharat et al., 2023), underreporting remains possible. Future studies could reduce reporting bias by combining self-reports with biochemical verification methods, such as expired carbon monoxide or cotinine, when feasible. Third, the one-month follow-up captures early cessation dynamics but does not allow conclusions about long-term abstinence. In addition, the potential influence of pharmacological smoking cessation treatments (e.g., nicotine replacement therapy) was not assessed and should be considered as a covariate in future research. Finally, findings are specific to patients with H&N cancer and may not generalize to other cancer populations or cultural contexts, highlighting the need for larger samples, longer follow-up periods, and cross-cultural replication.

## 5. Conclusions

To our knowledge, this is the first study to examine tobacco cessation after a cancer diagnosis through the lens of the HAPA. The findings suggested that volitional constructs, and particularly Action Control Efficacy, were more decisive than motivational factors in predicting short-term cessation outcomes. In addition, Coping Planning was inversely associated with both cigarette dependence and craving, underscoring the protective role of planning strategies. These results highlight the importance of targeting volitional resources early in the cessation process, beyond motivational factors alone, in order to improve intervention effectiveness. Future research should replicate these findings with larger samples, longer follow-up periods, and biochemical verification of smoking status to better understand how volitional mechanisms can sustain long-term abstinence in oncology populations.

## Figures and Tables

**Table 1:** Demographic characteristics of the sample (*N* = 89).

Mean Age, Years (SD)	59.6 (10.4)	
	*n*	%
Gender		
Female	23	25.8
Male	66	74.2
Marital status		
Single	48	53.9
In relationship	41	46.1
Socio-professional status		
Executives and intellectual professions	7	7.9
Self-employed and entrepreneurs	6	6.7
Employees and manual workers	23	25.8
Inactive population	53	59.6
Cancer diagnosis		
Oral cavity cancers	34	38.2
Oropharyngeal cancers	20	22.5
Laryngeal cancers	19	21.3
Hypo- and nasopharyngeal cancers	11	12.4
Other head and neck cancer	5	5.6
Cancer treatment		
Surgery	44	40.7
Radiotherapy	21	19.4
Chemoradiotherapy	17	15.7
Surgery and radiotherapy	20	18.5
Other treatments	6	5.6
Tumor stage		
Stage I/II	14	17.5
Stage III/IV	66	82.5
Number of cigarettes smoked daily T0		
2–10	54	60.7
11–20	27	30.3
21–30+	8	9
Number of cigarettes smoked daily T1		
0 cigarette	40	44.9
2–10	28	31.6
11–20	18	20.2
21–30+	3	3.3

**Table 2:** Pearson’s correlation between variables (*N* = 89).

Variables	1	2	3	4	5	6	7	8	9
1 Cigarette dependence T0	-								
2 Cigarette dependence T1	0.63 **	-							
3 Tobacco craving T0	0.68 **	0.50 **	-						
4 Tobacco craving T1	0.58 **	0.71 **	0.60 **	-					
5 Risk Perception T0	0.02	−0.04	−0.02	0.06	-				
6 Outcome Expectancies T0	0.15	0.13	0.08	0.10	0.35 **	-			
7 Recovery Self-Efficacy T0	−0.39 **	−0.34 **	−0.36 **	−0.29 **	0.13	−0.01	-		
8 Behavioral Intention T0	−0.08	−0.15	−0.09	−0.02	0.59 **	0.44 **	0.18	-	
9 Coping Planning T0	−0.28 **	−0.34 **	−0.26 *	−0.27 *	0.30 **	0.23 *	0.42 **	0.38 **	-
10 Action Control Efficacy T0	−0.35 **	−0.39 **	−0.39 **	−0.36 **	0.45 **	0.30 **	0.52 **	0.59 **	0.61 **

Note: * *p* < 0.05, ** *p* < 0.01.

**Table 3:** Hierarchical multiple linear regression predicting the score of cigarette dependence (*N* = 89).

	*β*	*t*	*p*	*R* ^2^	Δ*R*^2^
Step 1				0.26	0.69
Gender	−0.07	−0.58	0.56		
Age	0.04	0.36	0.72		
Marital status	−0.14	−1.19	0.24		
Socio-professional status	0.15	1.26	0.21		
Cancer diagnosis	−0.16	−1.39	0.17		
Cancer treatment	0.01	0.06	0.95		
Tumor stage	−0.06	−0.50	0.62		
Step 2				0.59	0.28
Gender	−0.09	−0.84	0.41		
Age	0.01	0.07	0.94		
Marital status	−0.20	−1.76	0.08		
Socio-professional status	0.16	1.37	0.17		
Cancer diagnosis	−0.04	−0.37	0.72		
Cancer treatment	−0.06	−0.55	0.59		
Tumor stage	0.04	−0.34	0.73		
HAPA Risk perceptions	0.34	2.92	**0.01**		
HAPA Outcome expectancies	0.26	2.02	**0.05**		
HAPA Recovery Self-efficacy	−0.07	−0.57	0.57		
HAPA Behavioral intention	−0.14	−0.92	0.36		
HAPA Coping planning	−0.20	−1.49	0.14		
HAPA Action Control Efficacy	−0.28	−1.67	0.10		

Note: Significant differences are in bold.

## Data Availability

The data supporting the findings of this study are publicly available on Zenodo at https://doi.org/10.5281/zenodo.17135975.

## Abbreviations

The following abbreviations are used in this manuscript:

H&N	Head and Neck
HAPA	Health Action Process Approach
